# Implementation of a web-based partnership support program for improving the quality of life of male patients undergoing infertility treatment: a pilot feasibility study

**DOI:** 10.1186/s13104-023-06431-x

**Published:** 2023-07-21

**Authors:** Kyoko Asazawa, Mina Jitsuzaki, Akiko Mori, Tomohiko Ichikawa, Masami Kawanami, Atsumi Yoshida

**Affiliations:** 1 Division of Nursing, Tokyo Healthcare University, 2-5-1 Higashigaoka, Meguro, Tokyo, 152-8558 Japan; 2 Department of Nursing, University of Occupational and Environmental Health, Fukuoka, Japan; 3 Division of Nursing, Shonan Kamakura University of Medical Sciences, Kanagawa, Japan; 4 Graduate School of Medicine, Chiba University, Chiba, Japan; 5 The Reproduction Center, Kiba Park Clinic, Tokyo, Japan

**Keywords:** Infertility, Male, Quality of life, Web browser

## Abstract

**Objectives:**

In this study, we aimed to implement and evaluate a Web-based partnership support program to enhance the QoL of male patients undergoing infertility treatment. We conducted a pilot study involving 41 infertile couples from September to October of 2021. We used a quasi-experimental design (pre-test and post-test with comparison) involving purposive sampling. A subgroup analysis was conducted to determine which demographics of the participants would benefit from the program.

**Results:**

Thirty-four participants (mean age 37.3 years; duration of infertility treatment 14.5 months) were included in the final analysis (follow-up rate 82.9%). Although there was no significant increase in the participants’ QoL under the Web-based partnership support program, the assisted reproductive technology group (*P* = 0.03), the no medical history group (*P* = 0.032), and the with experience of changing hospital group (*P* = 0.027) showed a significant increase in the relational subscale scores of the QoL before and after the program. The majority of the participants (n = 29; 85.3%) expressed satisfaction with the support program. Participation in the Web-based partnership support program may improve the QoL of some men undergoing infertility treatment.

*Trial registration* Retrospectively registered at the University Hospital Medical Information Network on 26 January 2023 (ID: UMIN0000 000050153).

## Introduction

Most developed countries are currently facing serious social problems owing to its declining birth rate and aging population [1–3]. In contrast, the number of couples suffering from infertility has been increasing, infertility is an enormous healthcare and social problem [4–8]. Patients undergoing infertility treatment have also been shown to have increased stress and poor quality of life (QoL) [9–12]. As infertility treatment is for both men and women, supportive interventions should target infertile couples at the dyad level, and include a component that enhances the marital function of the couples [13–15]. However, as there are very few programs that are unique to men, they are less likely to seek infertility care services than women [16]. Enhancing the partnership of infertile couples may lower their distress and maintain their QoL [17, 18]. There are not so many studies on interventions for male infertility patients [19–21].

Temporary social distance and closure of emergency medical services have been advocated to mitigate the adverse effects of the COVID-19 pandemic [22, 23]. As infertility patients have a higher level of distress, it is imperative to offer emotional support to reduce their stress and concerns [24–26]. However, to our knowledge, there is still no Web-based care program specifically developed for men receiving infertility treatment in Japan. In the present study, we aimed to implement and evaluate a Web-based partnership support program for enhancing the QoL of male patients undergoing infertility treatment. A subgroup analysis was planned to determine which demographics of the participants would benefit from the program.

## Main text

### Methods

#### Participants and procedures

The present non-randomized controlled trial was conducted among 41 infertile couples in Japan from September to October of 2021 at a leading fertility clinic in Japan. We used a quasi-experimental design for the pre-test and the post-test with comparison using purposive sampling. Potential participants were couples referred to the clinic for infertility treatment. The inclusion criteria were as follows: (i) must be within 3 years of the infertility treatment, (ii) have an online communication environment using a personal computer or smartphone at their home, and (iii) participation in the program as a couple. The exclusion criterion was the presence of any sexual dysfunctions to avoid additional mental burden. The sample size was calculated to be 35 participants using the software G*Power 3.1.9.7 with a one group nonparametric test, effect size d = 0.5, significance level (*α*) = 0.05, and power (1-*β*) = 0.8. Before the study, the participants were informed both verbally and in writing about the research goals and the confidentiality of any disclosed information. Subsequently, written informed consent was obtained from all the participants.

#### Intervention

Each participant was asked to reply to the self-reported questionnaire online using a personal computer or smartphone. All participants attended the program which was in the form of Web-based e-learning with the purpose of providing information about couple cooperation in the treatment stage. The interventions were couple-based and the surveys were conducted among the male participants only. An existing partnership support program for couples undergoing infertility treatment was modified to fit our Web-based partnership support program [27]. The original program included a number of small group sessions of 60 min, whereas the novelty of our present program was the development Web-based self-paced courses. This partnership support program consisted of information provision and a discussion between the couples. More specifically, it was designed such that the informational presentation would be watched for about 30 min and the discussion between couples would last for about 10 min. The information provision covered topics such as (i) gender differences and stress in infertility treatment, (ii) male and female emotions during infertility treatment, (iii) couple cooperation in the treatment stage, (iv) information related to the pregnancy test, and (v) communication techniques through video presentations. For the support program, a range of slides, stream of a video online, and practice forms were used. During the discussion, with the use of a dedicated communication form, the couples first described their feelings and thoughts about children and about their treatment. Subsequently, the couples exchanged their thoughts and feelings. The communication form-based discussions were continued at home for more than 10 days during the 2 week period. This program lasted 40 min per session and the frequency was once a day for 10 days. The post-test evaluation was set after 2 weeks to avoid discontinuance of the infertility treatment because of a partner’s pregnancy. It was assumed that the couples would have the opportunity to make the most of their partnership within 2 weeks. However, the duration of the program was set because it was determined that accurate evaluation of the program would not be possible if the participants’ psychological situation changed due to their partners' pregnancies.

#### Measures

The outcome measures included QoL and distress. A program evaluation was conducted among the participants using a 5-item self-developed questionnaire. The survey evaluated the participants’ opinions on program comprehension, program satisfaction, program availability, match of expectations, and ease of viewing the site. Additionally, we asked the participants to provide their opinions regarding the program by writing freely.

The FertiQoL tool developed by Boivin and colleagues (2011) [28] was used for evaluating the QoL of men in terms of their personal experiences with fertility problems. This tool has been translated into 46 languages and includes 6 subscales, namely, emotional, mind/body, relational, social, environment, and tolerability. FertiQoL consists of 34 items with 5 response categories ranging from 0 (lower QoL) to 4 (higher QoL). A higher score on the total FertiQoL scale or one of the subscales (range 0–100) indicates a better QoL. Boivin and colleagues (2011) [28] reported the Cronbach’s alpha coefficient of FertiQoL to be in the range of 0.72–0.92. The construct validity of the English version of FertiQoL was confirmed using item analysis and exploratory factor analyses by the developers [28]. In the present study, we used the Japanese version of FertiQoL. The overall Cronbach’s alpha of the Japanese version of FertiQoL was 0.92 and ranged from 0.67 to 0.86 in the 6 subscales [29].

The Japanese version of the distress scale developed by Asazawa and Mori (2015) [18] was used to evaluate the psychological distress of infertile couples. This distress scale consists of the following 3-item inventory: (i) “Do you feel stressed by the treatment?” (ii) “Do you feel depressed because of the treatment?” and (iii) “Do you have anxiety from the treatment?” The response categories range from 1 (strongly disagree) to 5 (strongly agree). Higher scores indicate the presence of a higher distress level. The Cronbach’s alpha coefficient was found to be 0.89 from the data of Japanese infertile couples [18]. Exploratory factor analysis was used to determine the construct validity of the distress [10]. Additionally, we asked the participants to provide their opinions regarding the program by writing freely.

### Data analysis

Data were analyzed using SPSS software (version 26.0). A pre-test and post-test comparison was carried out using a non-parametric test as the scales were not normally distributed according to the Shapiro–Wilk test. Pre-intervention and post-intervention changes in the participants were analyzed using the Wilcoxon signed-rank test. A subgroup analysis was conducted by attribute to determine which demographics of the participants would benefit from the program. A frequency distribution table was created from the 5 items of the process evaluation. A *P*-value of < 0.05 indicated a statistically significant difference. The open-ended remarks were analyzed using constant comparative analysis.

## Results

**Fig.1:**
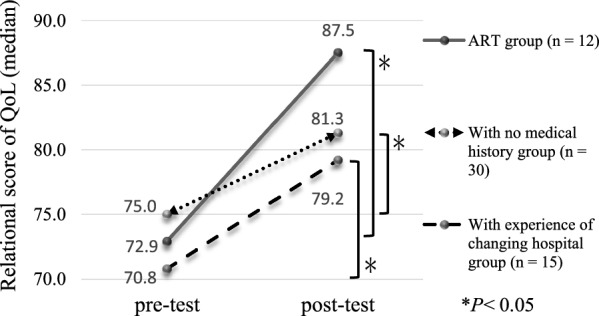
Comparison of the relational subscale scores between pre-test and post-test among participants in the ART, with no medical history, and with experience of changing hospital groups. *Wilcoxon signed-rank test

Of 43 potential candidates, 41 met the inclusion criteria and recruited in the study. The final analysis included 34 participants (response rate, 82.9%). Based on the test results, there were no significant differences in the pre-test and post-test scores in the 2 scales (i.e., QoL and distress) (Table 1). QoL and distress scores were comparatively tested before and after the intervention for each attribute subgroup. The assisted reproductive technology (ART) group and non-ART group were divided and analyzed. In the ART group, the post-test relational subscale score (median = 87.5, IQR = 76.0–91.7) was significantly higher than the pre-test relational subscale score (median = 72.9, IQR = 63.5–83.3) (*P* = 0.03). The analyses were divided into 2 groups: with a medical history group and with no medical history group. In the with no medical history group, the post-test relational subscale score (median = 81.3, IQR = 70.8–87.5) was significantly higher than the pre-test relational subscale score (median = 75.0, IQR = 66.7–83.3, *P* = 0.032). The analyses were divided into a group with experience of changing hospital and a group without experience of changing hospital. In the group with experience of changing hospital, the post-test relational subscale score (median = 79.2, IQR = 58.3–87.5) was significantly higher than the pre-test relational subscale score (median = 70.8, IQR = 54.2–79.2, *P* = 0.027) (Fig. 1).

**Table 1:** Demographic characteristics of the participants and comparison between pre-test and post-test of each scale

Participants’ attributes	Median(IQR)	
Age (years)^a^	37.0 (33.0–42.0)	
Duration of marriage (months)^a^	35.0 (18.0–59.0)	
Duration of infertility (months)^a^	24.0 (15.0–38.8)	
Duration of infertility treatment (months)^a^	12.0 (4.0–21.8)	
Marital status^b^		
First marriage	29 (85.3)	
Remarried	4 (11.8)	
Common-law marriage	1 (2.9)	
Children^b^		
Yes	1 (2.9)	
No	33 (97.1)	
Significant medical history^b^		
Yes	4 (11.8)	
No	30 (88.2)	
Causes of infertility^b^		
Unexplained	10 (29.4)	
Male factor	15 (44.1)	
Female factor	4 (11.8)	
Male and female factors	5 (14.7)	
Type of treatment ^b^		
Under consideration	3 (8.8)	
Timing of therapy	4 (11.8)	
Ovulation-inducing drugs	1 (2.9)	
Artificial insemination	14 (41.2)	
Assisted reproductive technology	12 (35.3)	
Experience of changing hospital^b^		
Yes	15 (44.1)	
No	19 (55.9)	
Pre-test FertiQoL tool^a^	72.1 (64.3–80.5)	^*^n.s
Post-test FertiQoL tool^a^	73.5 (64.0–76.5)	
Pre-test Distress scale^a^	7.5 (4.0–12.0)	^*^n.s
Post-test Distress scale^a^	9.0 (5.8–12.0)	

Data are expressed as ^a^Median (IQR) or ^b^number (%)

*IQR* interquartile range, *n.s.* not significant

^*^Wilcoxon signed-rank test

**Fig. 2:**
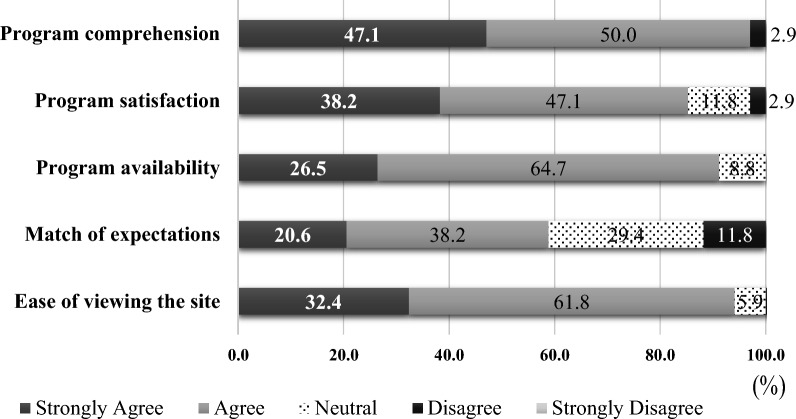
Evaluation process of the participants

Based on the participants’ response, 33 (97.1%) comprehended the program, 29 (85.3%) of whom were satisfied with the treatment methods, 31 (91.2%) were positive regarding program availability, 20 (58.8%) indicated a high match between their expectations and the implementation, and 32 (94.2%) felt the ease of viewing the site (Fig. 2). The content analysis of the open-ended responses revealed 3 categories: (1) gained information, (2) realized the importance of communication, and (3) needed minor modifications.

## Discussion

This study demonstrated that the male patients undergoing infertility treatment in the ART with no medical history group and with experience in changing hospital group showed improvement in the relational subscale of QoL. Additionally, the high satisfaction for the program and the availability of the program were judged to be beneficial from the standpoint of the care recipient. However, this program for men undergoing infertility treatment showed no significant effect on QoL and distress. This indicates that participation in the program had modest beneficial effects. These findings should be considered as incremental benefits at this point and can be used as inclusion/exclusion criteria for the next study.

The 2 factors underlying the lack of a significant effect of the program on QoL and distress were the wide range of selection criteria for participants and the lack of individual support. Patients with male factor and those on ART treatment reportedly have a lower QoL than other patients [10]. Participant selection criteria should have been set to having a male factor and for the treatment stage to be currently on ART treatment or just before ART treatment. In a previous study, the factors responsible for the low QoL were length of infertility, male factors, and poor spouse support [10, 30, 31]. Therefore, nursing care is necessary to reduce the decline in their QoL.

The responses of the participants indicated a high level of satisfaction for the program, as well as the availability and easy comprehension of the program. The participants also felt the ease of viewing the sites. Online psychoeducational support was reported to reduce stress and increase self-efficacy of women [32, 33]. Our Web-based support program targets male patients and is considered valuable at a time when face-to-face support is difficult with the persistence of COVID-19 infection. In addition, this support program is free and can be viewed as many times as needed, making it easy for patients to take advantage of the program in terms of time and cost. However, the match of expectations was low at 58.8%. 35.3% of the participants were in the ART phase of treatment, suggesting that they already understood the content of the program. Careful consideration should be given to the appropriate time to participate in the program, as it is better to target patients in the pre-ART stage to prevent a decrease in QoL.

## Conclusion

We developed a Web-based partnership support program for men undergoing fertility treatment. Although this program showed no significant changes in the QoL and distress, the participants in the ART treatment group, with no medical history group, and with experience of changing hospital group showed a significant increase in the relational subscale of QoL. Moreover, most of the participants were satisfied with the content of the program, felt that some of the contents can be improved and used in the future, and acknowledged the need for such a Web-based intervention program. A further enhanced program modified based on the needs identified and participants’ feedback would provide cost-effective and beneficial support to men undergoing fertility treatment and the couple.

## Limitations

As the couples were recruited from a high-level infertility treatment facility which was capable of addressing both male and female factors, the couples’ background may be different from that of the average Japanese couples. There is insufficient confirmation whether the couples have engaged in a helpful discussion.

## Data Availability

All data generated or analysed during this study are included in this published article.

## Abbreviations

QoL: Quality of life
COVID-19: COronaVirus Infectious Disease, emerged in 2019
ART: Assisted Reproductive Technology

## References

[1] Ministry of Health, Labour and Welfare. Changes in the number of births. 2021 (in Japanese). https://www.mhlw.go.jp/toukei/saikin/hw/jinkou/tokusyu/syussyo07/dl/01.pdf. Accessed 24 December 2021.

[2] United Nations. Demographic and Social Statistics 2022. https://unstats.un.org/unsd/demographic-social/products/vitstats/index.cshtml. Accessed 10 December 2022.

[3] Nomura K, Karita K, Araki A, Nishioka E, Muto G, Iwai-Shimada M (2019). For making a declaration of countermeasures against the falling birth rate from the Japanese society for hygiene: summary of discussion in the working group on academic research strategy against an aging society with low birth rate. Environ Health Prev Med.

[4] Martinez G, Daniels K, Chandra A (2012). Fertility of men and women aged 15–44 years in the United States: national survey of family growth, 2006–2010. Natl Health Stat Report.

[5] Ishihara O, Jwa SC, Kuwahara A, Katagiri Y, Kuwabara Y, Hamatani T (2020). Assisted reproductive technology in Japan: a summary report for 2018 by the ethics committee of the japan society of obstetrics and gynecology. Reprod Med Biol.

[6] Adamson GD, de Mouzon J, Chambers GM, Zegers-Hochschild F, Mansour R, Ishihara O (2018). International committee for monitoring assisted reproductive technology: world report on assisted reproductive technology, 2011. Fertil Steril.

[7] Centers for Disease Control and Prevention. Infertility FAQs.2022. https://www.cdc.gov/reproductivehealth/infertility/index.htm. Accessed 10 December 2022.

[8] Beaujouan E (2020). Latest-late fertility? Decline and resurgence of late parenthood across the low-fertility countries. Popul Dev Rev.

[9] Palomba S, Daolio J, Romeo S, Battaglia FA, Marci R, La Sala GB (2018). Lifestyle and fertility: the influence of stress and quality of life on female fertility. Reprod Biol Endocrinol.

[10] Asazawa K, Jitsuzaki M, Mori A, Ichikawa T, Shinozaki K, Porter SE (2019). Quality-of-life predictors for men undergoing infertility treatment in Japan. Jpn J Nurs Sci.

[11] Warchol-Biedermann K (2021). The etiology of infertility affects fertility quality of life of males undergoing fertility workup and treatment. Am J Mens Health.

[12] Rashidi B, Montazeri A, Ramezanzadeh F, Shariat M, Abedinia N, Ashrafi M (2008). Health-related quality of life in infertile couples receiving IVF or ICSI treatment. BMC Health Serv Res.

[13] Ying L, Wu LH, Loke AY (2016). The effects of psychosocial interventions on the mental health, pregnancy rates, and marital function of infertile couples undergoing in vitro fertilization: a systematic review. J Assist Reprod Genet.

[14] Galhardo A, Cunha M, Pinto-Gouveia J (2013). Mindfulness-based program for infertility: efficacy study. Fertil Steril.

[15] Frederiksen Y, O'Toole MS, Mehlsen MY, Hauge B, Elbaek HO, Zachariae R (2017). The effect of expressive writing intervention for infertile couples: a randomized controlled trial. Hum Reprod.

[16] Anderson JE, Farr SL, Jamieson DJ, Warner L, Macaluso M (2009). Infertility services reported by men in the United States: national survey data. Fertil Steril.

[17] Asazawa K, Jitsuzaki M, Mori A, Ichikawa T, Shinozaki K (2018). Supportive care needs and medical care requests of male patients during infertility treatment. Open J Nurs.

[18] Asazawa K, Mori A (2015). Development of a partnership causal model for couples undergoing fertility treatment. Jpn J Nurs Sci.

[19] Asazawa K, Jitsuzaki M, Mori A, Ichikawa T, Shinozaki K (2020). Effectiveness of a spousal support program in improving the quality of life of male patients undergoing infertility treatment: a pilot study. Int J Community Based Nurs Midwifery.

[20] Bisht S, Banu S, Srivastava S, Pathak RU, Kumar R, Dada R (2020). Sperm methylome alterations following yoga-based lifestyle intervention in patients of primary male infertility: a pilot study. Andrologia.

[21] Monirian F, Khodakarami B, Tapak L, Kimiaei Asadi F, Aghababaei S (2022). The effect of couples coping enhancement counseling on stress and dyadic coping on infertile couples: a parallel randomized controlled trial study. Int J Fertil Steril.

[22] ASRM. American Society for Reproductive Medicine (Asrm) Patient Management and Clinical Recommendations during the Coronavirus (Covid-19) Pandemic. https://www.asrm.org/news-and-publications/covid-19/statements/patient-management-and-clinical-recommendations-during-the-coronavirus-covid-19-pandemic/. 2020. Accessed 24 December 2021.

[23] Alviggi C, Esteves SC, Orvieto R, Conforti A, La Marca A, Fischer R (2020). COVID-19 and assisted reproductive technology services: repercussions for patients and proposal for individualized clinical management. Reprod Biol Endocrinol.

[24] Biviá-Roig G, Boldó-Roda A, Blasco-Sanz R, Serrano-Raya L, DelaFuente-Díez E, Múzquiz-Barberá P (2021). Impact of the COVID-19 pandemic on the lifestyles and quality of life of women with fertility problems: a cross-sectional study. Front Public Health.

[25] Ben-Kimhy R, Youngster M, Medina-Artom TR, Avraham S, Gat I, Marom Haham L (2020). Fertility patients under COVID-19: attitudes, perceptions and psychological reactions. Hum Reprod.

[26] Patel A, Sharma PSVN, Kumar P (2018). Role of mental health practitioner in infertility clinics: a review on past, present and future directions. J Hum Reprod Sci.

[27] Asazawa K (2015). Effects of a partnership support program for couples undergoing fertility treatment. Jpn J Nurs Sci.

[28] Boivin J, Takefman J, Braverman A (2011). The fertility quality of life (FertiQoL) tool: development and general psychometric properties. Hum Reprod.

[29] Asazawa K, Jitsuzaki M, Mori A, Ichikawa T, Shinozaki K, Yoshida A (2018). Validity and reliability of the Japanese version of the fertility quality of life (FertiQoL) tool for couples undergoing fertility treatment. Open J Nurs.

[30] Sarafraz Yazdi M, Nasiri R, Gharaei Jomei M, Sarafraz YS (2020). Quality of life and general health in pregnant women conceived with assisted reproductive technology: a case-control study. Int J Fertil Steril.

[31] Namavar Jahromi B, Mansouri M, Forouhari S, Poordast T, Salehi A (2018). Quality of life and its influencing factors of couples referred to an infertility center in Shiraz. Iran Int J Fertil Steril.

[32] Cousineau TM, Green TC, Corsini E, Seibring A, Showstack MT, Applegarth L (2008). Online psychoeducational support for infertile women: a randomized controlled trial. Hum Reprod.

[33] Park J, Shin N (2021). Development and application of a web-based integrated support service program for infertile women. Inquiry.

